# In Memoriam—Dr. L. B. Wells

**Published:** 1894-05

**Authors:** 


### IN MEMORIAM—DR. L. B. WELLS.

At 3.30 in the afternoon of Friday, March 23d, 1894, died the venerable physician, Dr. Lucien B. Wells, at his home in the city of Utica, New York, in his eighty-fourth year. This event follows an illness of about one week.

One who was well-nigh a perfect illustration of good citizenship has passed away. In his family relations, in his long career as a physician, bringing healing and comfort to the sick, in his relation to the church with which he was so long identified—in these and in his contact with his fellow-men there went to his credit daily the esteem and confidence and affectionate regard awarded to just and true and conscientious living.

Dr. Wells was born in a historic town—the town of Pompey,

Onondaga County, in the State of New York, on the 8th day of October in the year 1810. He came of patriotic stock. His grandfather on the paternal side was Colonel Levi Wells, of Colchester, Conn., who served in the Revolutionary war from its commencement to its close, first as a captain, and finally as a colonel. He was a fearless soldier and fought in seventeen battles, in one of which he was taken prisoner. His room-mate in captivity for a time was the celebrated Ethan Allen. It was Colonel Wells's youngest son Asa who was the father of Dr. Wells. Asa came to Pompey from Connecticut in 1803, and became a prominent citizen. He was at first a farmer and a surveyor, but he was soon a Justice of the Peace, afterward a County Judge, and then a member of the State Legislature. It was perhaps equally to his honor that he was one of the founders of the famous Pompey Academy, where many noted men have received considerable parts of their respective educations. The wife of Asa and the mother of Dr. Wells was Chloe Hyde, of Ellington, Conn. The Hyde family was distinguished for character as well as for longevity. The blood of the Hydes courses to-day in the veins of President Grover Cleveland. Chloe Hyde Wells lived to see the beginning of her ninety-third year, while one of her brothers lived to the age of ninety-six.

It was from such stock that Dr. Wells came. His life was destined to illustrate fairly the best qualities of his ancestry. He was educated at Pompey Academy. Then he entered upon the study of medicine with Dr. Pomeroy and Dr. J. P. Batchelder, in Utica, and when he graduated from Fairfield Medical College, in 1832, he was well-equipped for the beginning of his professional life. For two years he remained in Utica with Dr. Batchelder. Then he returned to his native county of Onondaga, practicing in Apulia and then in Pompey. He was then a physician of the old or regular school. In 1846, his attention was drawn to Homœopathy. He had become dissatisfied with the principles of the old school, or with what he deemed "its contradictions and uncertainties," and he had even thought of abandoning the profession and seeking other means of securing a livelihood. In the theory and principles of Hahnemann he found that which satisfied his conscience and renewed his devotion to his profession. As soon as his adoption of the new system was announced, he and four other members of the Onondaga County Medical Society were summoned to give an account of their alleged apostacy. This is a copy of the notice which Dr. Wells received:

"You are hereby notified to appear at a special meeting of the Onondaga County Medical Society, to be held at the Syracuse House in the city of Syracuse on Tuesday, the 10th day of September next, at 10 o'clock A. M., to show cause why you should not be expelled from said society for preaching Homœopathy, which by this society is deemed *quackery*.

"ABRAM HAHN,

"SYRACUSE, April 28th, 1847. "*Secretary*."

Of the accused only Dr. Wells appeared to meet the accusation, but he found no allopathic physician present to press the charge. It subsequently appeared that having consulted counsel, his accusers had concluded to let the matter drop. His associates who failed to appear were not cowards—some of them attained eminence in their profession—but they elected to pursue another course: they voluntarily withdrew from the Society, and Dr. Wells soon after followed their example.

It was in 1850 that Dr. Wells returned to take up his residence in Utica. For a time he was associated with Dr. Frederick Humphreys, who afterward went to New York. A later partner was Dr. Thomas F. Pomeroy, a brother of Theodore Pomeroy, Esq., of Utica, and a son of Dr. Theodore Pomeroy, with whom Dr. Wells commenced his medical studies. This partnership existed from 1853 to 1857, when Dr. Pomeroy removed to Detroit, where he practiced for many years until failing health compelled him to retire. An obituary notice of Dr. T. F. Pomeroy was published in THE HOMŒOPATHIC PHYSICIAN for May, 1892, at page 216.

After the severance of the partnership with Dr. Pomeroy,

Dr. Wells practiced alone, but always kept up a correspondence with his old partner.

Dr. Wells' practice was on the line of the strictest adherence to the principles of Hahnemann. Though he lived to see the most remarkable and widely practiced innovations upon the method of Hahnemann, yet never did he swerve from the plain principles and practice of the law of the similars. He was eminently successful in his practice and widely known by a large number of patients who were devotedly attached to him. As his years advanced, these were reluctant to place themselves under other ministrations and made their friendly demands upon his services down almost to the day that his fatal illness began.

It was a part of the character of Dr. Wells that he should have a church connection to which he should be constant and devoted. For nearly half a century he has been one of the pillars of Westminster Church in Utica, enjoyed its privileges, and rejoiced in its spiritual even more than in its temporal prosperity, and contributed to its Christian and benevolent spirit. His religious light was a lamp that burned steadily. For forty years he has been one of the Elders of Westminster Church. He was for twenty-nine years Treasurer of the Session and only last year relinquished these duties on account of his advancing years. The occasion was marked by the adoption of a minute warmly acknowledging his services, and this was certified with the gift of a beautiful copy of *Ben-Hur* as a further mark of the gratitude and esteem of his associates.

Dr. Wells was married on the 2d day of October, 1837, in Southampton, Mass., to Miss Orissa M. Searl. The golden anniversary of this happy and fortunate union was celebrated in 1887, and was the occasion of many appropriate felicitations. In all that Dr. Wells was and did Mrs. Wells has been a sharer and helpmeet. Only He who knows all can measure the good that becomes a part of men when such wives are their counselors and supporters and sympathizers. Mrs. Wells survives him together with their only son, Edward H. Wells, a lawyer. There is but one other survivor of the Doctor's immediate relatives. He was one of a family of eleven children, four sons and seven daughters. All lived to maturity and some have died in advanced years. But Mrs. Morris Beard, of Pompey Hill, now only remains.

It is believed that Dr. Wells was the oldest living homœopathic physician in the United States. Readers of this journal will remember that this subject was discussed in these pages in the number for January, 1892, page 31, and March, 1892, page 113. He was once the President of the State Homœopathic Society, and has long been an honored member of the American Institute of Homœopathy and the International Hahnemannian Association.

To *The Utica Daily Observer*, of March 23d, the editor of this journal is indebted for several copious extracts which have been incorporated in the present article.

Says Dr. Isaac Dever, of Clinton, N. Y., in a letter to the editor, commenting upon the *Observer's* obituary: "As a man and citizen Dr. Wells was upright and just. As a friend he was open-hearted, free, and ever ready to lend a helping hand. As a homœopathist he was loyal to the letter of the law. Last week, on the fifteenth of March, he attended the Central New York Homœopathic Society meeting at Syracuse. He read a paper and took an active part in the papers and discussions and expressed himself as better in health than he had been for years. What a glorious termination of a well-rounded professional life! I loved him!"

